# Multimodal sequence dynamics and convergence optimization in dual-stream LSTM networks for complex physiological state estimation

**DOI:** 10.3389/fnbot.2026.1760494

**Published:** 2026-02-06

**Authors:** Xiaoxiao Cao

**Affiliations:** Department of Public Physical Education, China Academy of Art, Hangzhou, Zhejiang, China

**Keywords:** attention mechanism, convergence analysis, multimodal dynamics, recurrent neural networks, sequence modeling, state estimation

## Abstract

**Introduction:**

The integration of virtual simulation with intelligent modeling is crucial for advancing the scientization and personalization of volleyball physical training. This study aims to overcome the convergence instability and feature misalignment in modeling multimodal kinematic and physiological sequences.

**Methods:**

A dynamical framework based on a Dual-Stream Long Short-Term Memory network integrated with a temporal attention mechanism is proposed. The framework decouples heterogeneous feature learning and optimizes temporal weight distribution.

**Results:**

Experimental validation on complex motion state estimation demonstrates that the proposed model reduces load modeling error to 3.8% and achieves a motion classification accuracy of 93.1%. The velocity trajectory fitting coefficient of determination is 0.91 with a peak deviation of 0.05 m/s.

**Discussion:**

These results confirm the effectiveness of the attention-based DS-LSTM in optimizing multimodal sequence modeling for training state estimation and feedback.

## 1 Introduction

As a sport that combines both competitive and entertaining elements, volleyball places high demands on athletes’ physical fitness (Albaladejo-Saura et al., 2023; Lin et al., 2024; Pawlik and Mroczek, 2023). Physical training not only determines an athlete’s explosive power, endurance, and agility during competition but also plays a crucial role in injury prevention and extending their athletic career (Esposito et al., 2024; Rebelo et al., 2022; Tan et al., 2023). In recent years, the development of virtual simulation technology has brought new opportunities to sports training. Virtual reality and augmented reality platforms, combined with sensor acquisition and intelligent analysis, can provide athletes with immersive training environments and data-driven feedback (Li et al., 2024; Sousa et al., 2023). Research on how to combine virtual simulation with intelligent modeling for volleyball physical training not only promotes innovation in training models but also has practical value in improving the scientific nature and personalization of training (Duan, 2021; Yunchao et al., 2023).

Current research on volleyball physical training faces three key challenges. First, motion capture and muscle load monitoring data often exhibit high dimensionality and temporal dependencies, making it difficult for traditional statistical methods to accurately characterize their dynamic patterns (Amin et al., 2023; Bae et al., 2024; Suo et al., 2024). Second, the data types collected by multimodal sensors vary widely, and asynchrony and noise exist between posture, muscle load, and velocity curves, impacting data fusion and modeling accuracy (Hafer et al., 2023; Tsanousa et al., 2022). Third, existing training feedback mechanisms largely rely on empirical judgment and lack real-time prediction and interpretable feedback based on neural network models, making personalized training optimization difficult to implement (Hribernik et al., 2022; Vec et al., 2024). These difficulties have hindered the practical application of virtual simulation technology in volleyball physical training.

To address these challenges, various technical approaches have been proposed (Putranto et al., 2023; Richlan et al., 2023). Convolutional neural networks have been used for motion recognition, but their structure is biased towards static feature extraction, making it difficult to capture the dynamic evolution of long time series (Huang and Cai, 2023; Le et al., 2022). Recurrent neural networks have demonstrated effectiveness in movement classification tasks (Amin and Noh, 2024), and possess certain advantages in time series modeling. However, they are susceptible to vanishing gradients in multimodal data fusion, resulting in insufficient convergence speed and stability (Su et al., 2025; Waqas and Humphries, 2024). Multi-sensor fusion methods have attempted to use weighted averaging and Kalman filtering, but their ability to fit complex nonlinear relationships is limited, making it difficult to accurately reflect the coupling effects between different training features (Tang et al., 2023; Urrea and Agramonte, 2021). Attention mechanisms have been introduced in some studies to highlight key features, but they are mostly used in single-modal scenarios and lack cross-modal interaction (Lu et al., 2023; Wang and Liang, 2023). Overall, these methods still have shortcomings in accurately modeling multimodal time series data and optimizing personalized training, and have not yet addressed the need for scientific and real-time feedback in volleyball physical training (Salim et al., 2024; Wang et al., 2022).

To address the shortcomings of traditional methods in multimodal time-series data modeling and training feedback, this paper proposes a dynamic modeling approach based on DS-LSTM and a temporal attention mechanism. This framework uniquely applies temporal attention after separate kinematic and physiological temporal encoding, rather than before fusion. This structured decoupling and reweighting of modalities enhances convergence stability and mitigates temporal misalignment. This approach first synchronizes and suppresses the temporal noise of posture, electromyography, and acceleration data. Then, in a dual-stream architecture, kinematic and physiological features are fed into recursive units to capture their internal dynamic patterns. Interactive feature fusion is then used to integrate cross-modal information. Furthermore, a temporal attention mechanism is introduced to automatically identify key performance-affecting periods during training and weight these features, ultimately outputting training status predictions and optimization recommendations. This approach enables dynamic modeling and intelligent feedback for volleyball physical training in a virtual simulation environment, enhancing the interpretability and convergence stability of training data and providing athletes with a personalized, scientific training path.

## 2 Methods

### 2.1 Data collection and preprocessing

In virtual simulation experiments of volleyball physical training, the acquisition of multimodal time-series data is a prerequisite for dynamic modeling. This paper constructs a dataset based on motion capture sensors, myoelectric sensors, and accelerometers. Motion capture sensors output the athlete’s joint positions and angles in three-dimensional space, reflecting the temporal changes in posture and movement. Myoelectric sensors record the electrophysiological signals of major muscle groups during training, revealing the muscle load under high-intensity exercise. Accelerometers measure the velocity and acceleration curves of training movements to characterize the explosive power and rhythmic characteristics of the movement. Due to differences in sampling rate, data dimension, and duration settings among the three sensor types, the raw data must be standardized. The data acquisition parameters are shown in Table 1.

**Table 1:** Data acquisition parameters.

Sensor type	Collected features	Sampling rate (Hz)	Data dimension	Duration per trial (s)	Output format
Motion Capture	Joint positions, angles	120	18	30	3D coordinate matrix
EMG Sensor	Muscle electrical signals	1,000	8	30	Voltage time series
Accelerometer	Velocity, acceleration	200	6	30	Vector time series
Gyroscope	Angular velocity	200	3	30	Three-axis sequence
Force Sensor	Ground reaction force	500	3	30	Force time series

The raw data will be affected by noise and drift during the acquisition process, and directly inputting the model will cause convergence difficulties. To this end, targeted preprocessing methods are used for different signals: the electromyographic signal is filtered by a Butterworth bandpass filter to remove low-frequency drift and high-frequency interference, the motion capture and acceleration signals are filtered by sliding average to eliminate jitter, and the mechanical data are filtered by low-pass filtering to reduce high-frequency noise. All signals are then normalized to eliminate dimensional differences so that data from different modes are comparable within the same range. Normalization uses the standard scaler method, as shown in Equation 1 (Sinsomboonthong, 2022).


$$x^{\prime }=\frac{\left(x-\mu \right)}{\sigma }$$ (1)

In Equation 1, x is the original data value, μ represents the mean of the feature dimension, σ represents its standard deviation, and $x^{\prime }$ is the normalized output. This process centers the data and scales it based on variance, which is more robust for handling potential out-of-range data during model application.

Because the sampling rates of various sensors vary, timing alignment is necessary on a unified time axis. This paper uses the electromyographic signal with the highest sampling rate as the time reference and employs linear interpolation to interpolate the lower-sampling-rate signals, synchronizing all data at the same time step. To verify the effectiveness of preprocessing, a comparative analysis of signal quality metrics before and after filtering and alignment was performed, including signal-to-noise ratio, timing alignment error, and stability index. The results are shown in Table 2.

**Table 2:** Comparison of data preprocessing effects.

Data type	SNR before filtering (dB)	SNR after filtering (dB)	Alignment error (ms)	Stability index (0–1)
Motion Capture	18.2	28.7	4.6	0.82
EMG Data	12.5	24.3	3.2	0.88
Velocity Data	15.7	26.8	5.1	0.85
Angular Velocity	14.3	25.6	4.9	0.81

### 2.2 DS-LSTM structure modeling

In the dynamic modeling of multimodal time series data, kinematic and physiological signals differ significantly. Previous dual-stream LSTM models commonly perform early or mid-level fusion without explicit temporal reweighting, which limits their ability to suppress redundant segments in long training sequences. Attention-based recurrent architectures reported in related studies typically operate on concatenated multimodal representations, leading to interference between heterogeneous dynamics during attention computation. By contrast, the proposed DS-LSTM with temporal attention performs attention weighting only after independent temporal encoding, ensuring that the attention distribution reflects modality-consistent temporal patterns rather than mixed feature responses. Posture capture sequences are characterized by high dimensionality, strong temporal dependencies, and dynamic continuity, while electromyographic and acceleration signals reflect the changing patterns of muscle load and exercise intensity. To avoid the coupling interference of single-stream networks when processing cross-modal features, this paper employs a DS-LSTM architecture to perform temporal modeling of kinematic and physiological features in independent branches, and then implements interactive fusion of cross-modal features at a high level.

The kinematic flow takes the coordinate sequence of the posture key points as input and recursively updates the hidden state through the LSTM unit $X^{k}=\{x_{1}^{k},x_{2}^{k}\ldots x_{T}^{k}\}$ expanded in time steps to capture the dynamic dependency between spatial displacement and joint motion. Let the input sequence be, where $x_{T}^{k}$ represents t the key point feature vector at the moment, and the recursive process of LSTM can be expressed as Lees et al. (2021):


$$h_{t}^{k},c_{t}^{k}=\text{LSTM}(x_{t}^{k},h_{t-1}^{k},c_{t-1}^{k})$$ (2)

In Equation 2, $h_{t}^{k}$ represents the hidden state of $c_{t}^{k}$ the kinematic flow at time t, t is the unit state, and the recursive function includes nonlinear updates of the input gate, forget gate, and output gate. The output sequence of this branch $\left\{h_{1}^{k},h_{2}^{k}\ldots h_{T}^{k}\right\}$ describes the dynamic structure of the action sequence.

The physiological flow takes the fusion vector of myoelectric and acceleration signals as input and focuses on the activation intensity of muscle groups and the load changes during exercise. Assuming the input sequence is $X^{p}=\{x_{1}^{p},x_{2}^{p}\ldots x_{T}^{p}\}$ , its recursive process is consistent with Equation 2, but the hidden state and unit state are recorded as and, respectively, $h_{t}^{p}$ . $c_{t}^{p}$ This branch is formed through feature modeling of time series expansion $\left\{h_{1}^{p},h_{2}^{p}\ldots h_{T}^{p}\right\}$ and is used to characterize the dynamic changes at the physiological level of exercise.

**Figure 1:**
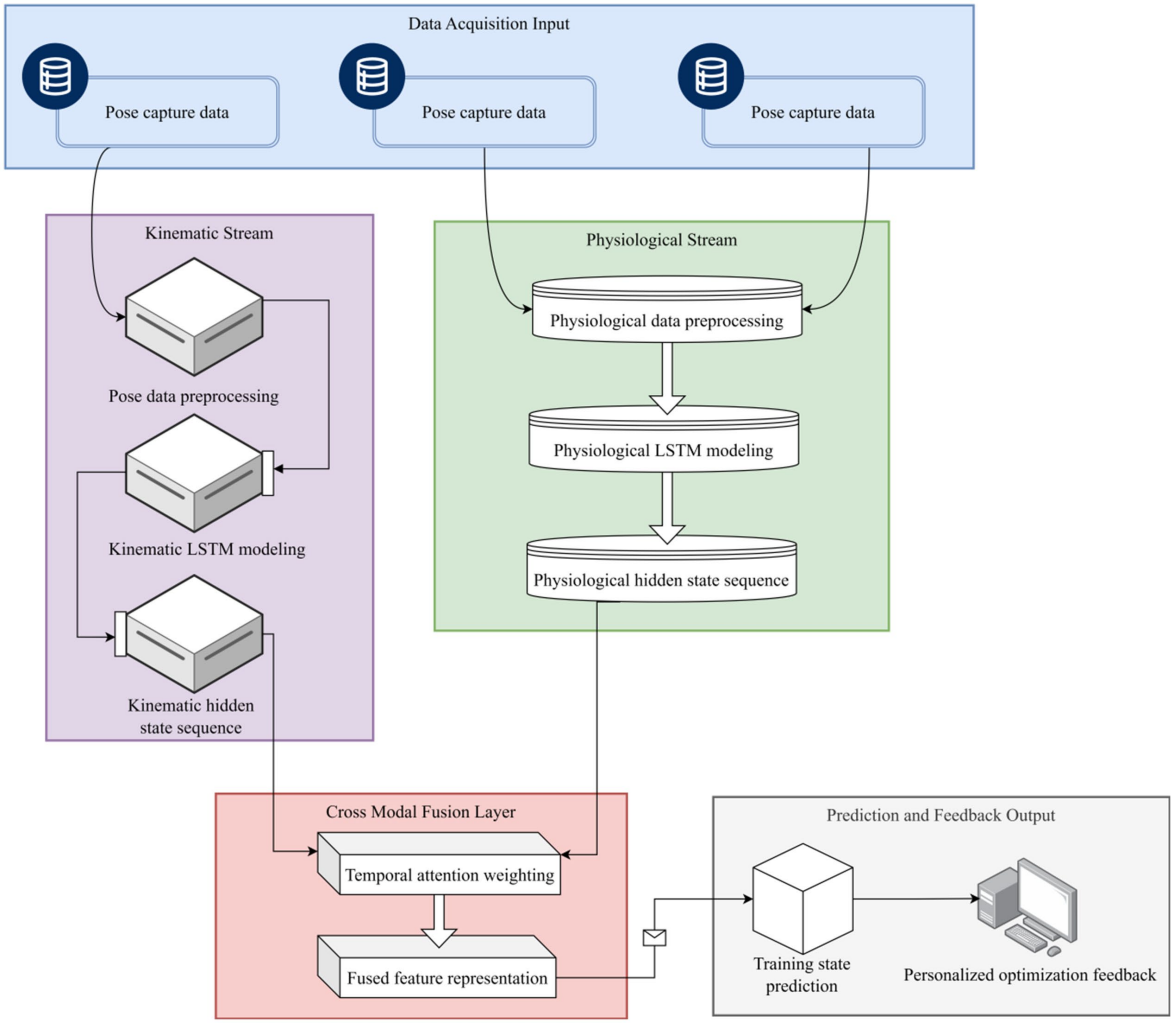
The proposed dual-stream LSTM (DS-LSTM) modeling framework. The architecture decouples kinematic (posture) and physiological (EMG/acceleration) data processing into independent streams. This ensures feature integrity before high-level fusion, allowing the model to capture distinct dynamic patterns for each modality.

In the dual-stream architecture, the kinematic and physiological streams maintain the integrity of temporal feature representation within independent recursive units, thus avoiding feature aliasing in early stages. High-level feature fusion will subsequently incorporate attention mechanisms and fully connected mapping to achieve cross-modal interaction modeling and prediction optimization. The overall framework of the dual-stream architecture is shown in Figure 1.

### 2.3 Temporal attention mechanism fusion

After the DS-LSTM architecture completes modeling of both kinematic and physiological features, a temporal attention mechanism is introduced to achieve weighted screening of key training segments. Multimodal time series data exhibits significant phase-by-phase differences during training. Features from some time periods contribute significantly to overall training state prediction, while others represent noise or redundant information. To address this issue, the hidden state sequences output by the DS-LSTM are set to be $H^{k}=\{h_{1}^{k},h_{2}^{k}\ldots h_{T}^{k}\}$ and $H^{p}=\{h_{1}^{p},h_{2}^{p},\ldots ,h_{T}^{p}\}$ , where $\;h_{t}^{k}$ and represent $h_{t}^{p}$ the kinematic and physiological flow feature vectors T at time t, respectively, t and is the total length of the sequence.

The calculation of attention weight is achieved through the interaction between the learnable parameter vector and the feature latent representation, and the weight distribution is defined as Hernández and Amigó (2021):


$$\alpha_{t}=\frac{\exp(u^{⊤}\tanh(Wh_{t}+b))}{\sum_{j=1}^{T}\;\exp(u^{⊤}\tanh(Wh_{j}+b))}$$ (3)

In Equation 3, $h_{t}$ represents the candidate feature vector after stacking and fusion of the dual-stream output, W and b is the trainable linear transformation parameter, and u is the global context vector. In Equation 3, the numerator is used to measure the moment t . The denominator ensures that the weights are normalized at all times to satisfy the constraints of the probability distribution.

The weights obtained $\alpha_{t}$ based on Equation 3 are used to weight the feature sequence to obtain the context representation after time series fusion:


$$\begin{array}{c} c=\sum_{t=1}^{T}\;\alpha_{t}h_{t} \end{array}$$ (4)

In Equation 4, c the temporal context vector integrates the dynamic contributions of key segments across modalities, eliminating interference from redundant features. In this process, training phases corresponding to moments with larger weights have a higher proportion in the final representation, ensuring that the model prioritizes high-value segments during prediction and feedback.

To further improve the effectiveness of cross-modal interaction, this paper introduces a feature fusion operation after the attention layer. The context vectors of kinematic and physiological features filtered by attention are spliced and nonlinearly mapped in the feature space:


$$\begin{array}{c} z=\sigma (W_{f}[c^{k};c^{p}]+b_{f}) \end{array}$$ (5)

Where, $c^{k}$ and are $c^{p}\;$ the attention-weighted contextual representations of the kinematic and physiological flows, respectively, represents $[c^{k};c^{p}]\;$ the vector concatenation operation, $W_{f}\;$ and are $\;b_{f}$ the fusion layer parameters, σ(·) and is a nonlinear activation function. Equation 5 implements cross-modal interactive expression, establishing an association between kinematic and physiological features in a shared space, ensuring that the final training state prediction not only relies on information from a single modality, but also integrates the interactive features of multimodal key time series segments.

**Figure 2:**
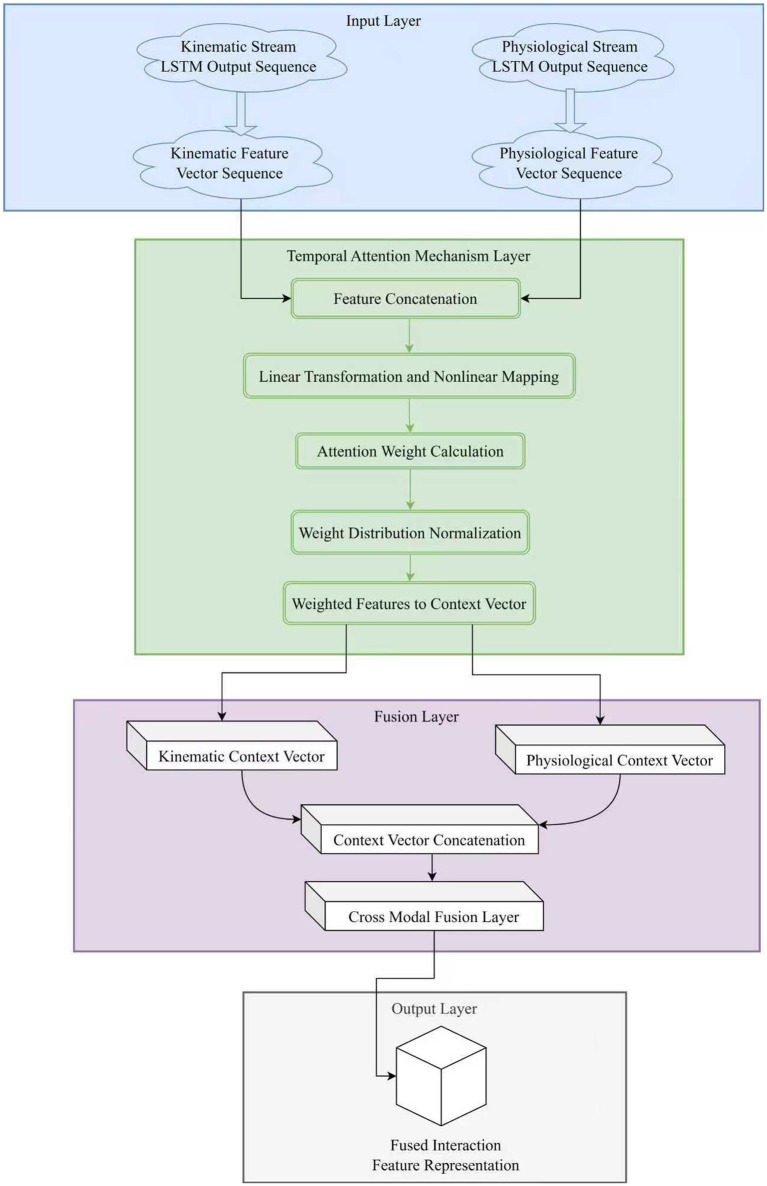
Schematic diagram of the temporal attention mechanism fusion structure. This layer calculates weight distributions for different time segments to filter key motion phases. It effectively integrates kinematic and physiological context vectors to generate a robust fused feature representation for state prediction.

While ensuring information selectivity, this structure also provides a more compact and discriminative input for the subsequent fully connected prediction layer, effectively improving the accuracy and stability of training optimization feedback. The overall process is shown in Figure 2.

### 2.4 Training optimization and feedback generation

The fused time series feature set $Z={\left\{z_{t}\right\}}_{t=1}^{T}$ Through a multi-layer feedforward network and task-specific output heads, the training objectives are mapped into posture prediction ${\hat{y}}^{\text{pose}}$ , electromyography prediction ${\hat{y}}^{\mathrm{emg}}$ , velocity prediction ${\hat{y}}^{\mathrm{vel}}$ , and attention weight sequences $A=\{a_{h,t}\}$ . The training objectives are uniformly expressed as a multi-task joint loss function, which is expressed as shown in Equation 6:


$$\begin{array}{l} L_{\text{total}}=w_{p}L_{\text{pose}}+w_{e}L_{\mathrm{emg}}+w_{v}L_{\mathrm{vel}}+\lambda_{\mathrm{att}}L_{\mathrm{att}}+\\ \lambda_{\text{smoot}h}L_{\text{smoot}h}+\lambda_{\mathrm{reg}}∥\theta {∥}_{2}^{2} \end{array}$$ (6)

Among them, $L_{\text{pose}}$ is the posture prediction error, $L_{\mathrm{emg}}$ is the electromyography regression error, $L_{\mathrm{vel}}$ is the velocity curve fitting error, $L_{\mathrm{att}}$ is the attention regularization term, $L_{\text{smoot}h}$ is the time series smoothing term, $\theta_{2}^{2}$ is the L2 regularization term of the model parameters; $w_{p},w_{e},w_{v}$ is the task weight, $\lambda_{\mathrm{att}},\lambda_{\text{smoot}h},\lambda_{\mathrm{reg}}$ is the regularization weight, and θ is the model parameter vector.

The posture loss is in the form of mean square error, which is defined as Equation 7.


$$L_{\text{pose}}=\frac{1}{N_{j}T}\sum_{t=1}^{T}\sum_{j=1}^{N_{j}}{\hat{y}}_{t,j}^{\text{pose}}-{y_{t,j}^{\text{pose}}}_{2}^{2}$$ (7)

Equation 7, $N_{j}$ is the number of joints, T is the number of time steps, ${\hat{y}}_{t,j}^{\text{pose}}$ is the model’s t Step j . The prediction of each joint $y_{t,j}^{\text{pose}}$ is the corresponding annotation.

The EMG regression loss is defined as Equation 8.


$$L_{\mathrm{emg}}=\frac{1}{N_{m}T}\sum_{t=1}^{T}\sum_{m=1}^{N_{m}}{\hat{y}}_{t,m}^{\mathrm{emg}}-{y_{t,m}^{\mathrm{emg}}}_{2}^{2}$$ (8)

Equation 8, $N_{m}$ is the number of myoelectric channels, ${\hat{y}}_{t,m}^{\mathrm{emg}}$ is the t Step m . The prediction of the channel $y_{t,m}^{\mathrm{emg}}$ is the labeled value.

The velocity fitting loss is defined as Equation 9:


$$L_{\mathrm{vel}}=\frac{1}{\mathrm{DT}}\sum_{t=1}^{T}{\hat{y}}_{t}^{\mathrm{vel}}-{y_{t}^{\mathrm{vel}}}_{2}^{2}$$ (9)

Equation 9, D is the velocity vector dimension, ${\hat{y}}_{t}^{\mathrm{vel}}$ is the model for the t . The prediction of $y_{t}^{\mathrm{vel}}$ the step speed is the true speed vector.

The attention regularization uses entropy terms to constrain the sparsity and interpretability of attention distribution, which is defined as follows:


$$L_{\mathrm{att}}=-\frac{1}{H}\sum_{h=1}^{H}\sum_{t=1}^{T}a_{h,t}\log\;(a_{h,t}+\varepsilon )$$ (10)

In H Equation 10, is the number of attention heads or channels, $a_{h,t}$ is the normalized attention weight (satisfies for each head $\sum_{t}a_{h,t}=1$ ), ε and is a numerical stabilization term to prevent logarithmic divergence.

In order to ensure the smoothness of the forecast time series, the first-order difference smoothing term is introduced, which is defined as follows:


$$L_{\text{smoot}h}=\frac{1}{T-1}\sum_{t=2}^{T}{\left\|{\hat{y}}_{t}-{\hat{y}}_{t-1}\right\|}_{2}^{2}$$ (11)

Equation 11 where ${\hat{y}}_{t}$ is the joint prediction vector obtained by splicing on each task, which is used to measure the continuity of predictions at adjacent moments.

In the mini-batch training framework, the batch loss is the average of samples within the batch, expressed as Equation 12:


$$L_{\text{batc}h}=\frac{1}{B}\sum_{i=1}^{B}L_{\text{total}}(x^{\left(i\right)})$$ (12)

Equation 12, B is the batch size, $x^{\left(i\right)}$ is the multimodal sequence and label of the i -th sample in the batch.

Backpropagation uses truncated backpropagation (BPTT) to expand to length in the time dimension τ . To suppress gradient explosion, the norm of the gradient is clipped. The clipping rule is as shown in Equation 13:


$$\tilde{g}=g\cdot \min(1,\frac{C}{∥g{∥}_{2}})$$ (13)

Equation 13, g is the unclipped gradient vector, $g_{2}\;$ is its two-norm, C is the clipping threshold, and $\tilde{g}$ is the clipped gradient.

The parameter update uses the Adam optimizer with bias correction, and the update process is based on Equation 14:


$$\begin{array}{rr} m_{t} & =\beta_{1}m_{t-1}+(1-\beta_{1})g_{t},\\ v_{t} & =\beta_{2}v_{t-1}+(1-\beta_{2})g_{t}⊙g_{t},\\ {\hat{m}}_{t} & =\frac{m_{t}}{1-\beta_{1}^{t}},\;\;{\hat{v}}_{t}=\frac{v_{t}}{1-\beta_{2}^{t}},\\ \theta_{t+1} & =\theta_{t}-\eta \frac{{\hat{m}}_{t}}{\sqrt{{\hat{v}}_{t}}+\varepsilon }. \end{array}$$ (14)

Equation 14, $g_{t}$ is the clipped gradient, $m_{t}$ and $v_{t}$ are the first-order and second-order moment estimates respectively, $\beta_{1},\beta_{2}$ is the momentum coefficient, ${\hat{m}}_{t},{\hat{v}}_{t}$ is the bias-corrected estimate, η is the learning rate, ε is the numerical stability term.

The cosine annealing learning rate scheduling is used, and t the learning rate during the training step is calculated according to Equation 15:


$$\eta_{t}=\eta_{\min}+\frac{1}{2}(\eta_{0}-\eta_{\min})(1+\cos(\mathrm{\pi t}/T_{\max}))$$ (15)

In Equation 15, $\eta_{0}$ is the initial learning rate, $\eta_{\min}$ is the minimum learning rate, $T_{\max}$ and is the maximum number of training steps; to be consistent with the convergence observation, take $T_{\max}=500$ .

When the validation set loss does not decrease in consecutive p=20 epochs, the training is terminated and the model parameters are rolled back to the lowest point of the validation set to retain the optimal performance.

The fused features are fed into the fully connected layer to generate personalized training feedback. Feedback generation forms a closed loop with four steps: feature mapping, deviation measurement, intensity mapping, and output mapping. The feedback feature vector is given by Equation 16:


$$f_{t}=\sigma (W_{f}z_{t}+b_{f})$$ (16)

In Equation 16, $W_{f}$ is the size $R_{f}\times D_{z}$ . The weight matrix is $b_{f}$ , the bias vector is $D_{z}$ , the fusion feature dimension is σ , the component-by-component Sigmoid activation function is used to limit the output to (0,1) the interval, and the feedback feature vector is $f_{t}$ .

The deviation metric uses the Euclidean deviation from the target baseline and is normalized within the window, defined as follows:


$$\Delta_{t}=y_{t}^{\mathrm{ref}}-{\hat{y}}_{t},\;\;s_{t}=\frac{{\left\|\Delta_{t}\right\|}_{2}}{\max_{t'\in W}{\left\|\Delta_{t'}\right\|}_{2}+\delta }$$ (17)

In Equation 17, $y_{t}^{\mathrm{ref}}$ is the target baseline vector, $\Delta_{t}$ is the deviation vector, ${\left\|\cdot \right\|}_{2}$ . is the Euclidean norm, and the window W . Used to calculate the normalized reference value, δ a small constant $s_{t}$ to prevent division by zero, and a normalized deviation scalar.

The deviation scalar is nonlinearly mapped to obtain the feedback strength $I_{t}$ , and the mapping is performed according to Equation 18.


$$I_{t}=\text{sigmoid}(\gamma \;s_{t})$$ (18)

In Equation 18, γ is the gain coefficient used to adjust the sensitivity of the deviation to intensity, sigmoid(·) is the standard S-shaped mapping, $I_{t}$ and is the scalar intensity.

The continuous control vector actually sent to the virtual simulation/teaching interface is obtained by linear mapping and clipping after splicing the feedback characteristics and intensity, and is expressed as follows:


$$u_{t}=\text{clip}(W_{u}[\begin{array}{c} f_{t}\\ {}[2\mathrm{pt}]I_{t} \end{array}]+b_{u},u_{\min},u_{\max})$$ (19)

In Equation 19, $W_{u}$ is the output mapping matrix, $b_{u}$ is the bias, $\text{clip}(\cdot ,\text{umin},\text{umax})\;\text{clip}(\cdot ,u_{\min},u_{\max})$ represents the component— $\left[u_{\min},u_{\max}\right]$ by-component clipping of the vector components by interval, $u_{t}$ and is the continuous feedback signal finally sent to the virtual simulation engine or the coaching end.

In order to make the generated feedback show a positive effect in the posterior evaluation, a closed-loop evaluation of the feedback utility is introduced and the evaluation signal is converted into an auxiliary loss for joint training, which can be rewritten as follows:


$$R_{t\to t+K}=\frac{1}{K}\sum_{i=1}^{K}\;(m_{t+i}-m_{t})$$ (20)

In Equation 20, $m_{t}$ is the scalar performance metric used to evaluate the training effect, K is the evaluation window length, $R_{t\to t+K}$ and is the average improvement within a given window.


$$L_{\mathrm{fb}}=-R_{t\to t+K},\;\;L{'}_{\text{total}}=L_{\text{total}}+\lambda_{\mathrm{fb}}L_{\mathrm{fb}}$$ (21)

In Equation 21, $\lambda_{\mathrm{fb}}$ is the feedback utility weight, $L{'}_{\text{total}}$ is the joint loss with feedback utility constraint, and is used to guide the model to generate feedback signals that reflect positive improvements in the posterior evaluation.

The training hyperparameters in this implementation are: batch size B=32 , truncation length τ=100 , gradient clipping threshold C=5.0 , Adam momentum coefficient $\beta_{1}=0.9,\beta_{2}=0.999$ , numerical stability term $\varepsilon =10^{-8}$ , initial learning rate $\eta_{0}=10^{-3}$ , minimum learning rate $\eta_{\min}=10^{-6}$ , maximum number of training steps $T_{\max}=500$ , early stopping window p=20 , attention regularization weight $\lambda_{\mathrm{att}}=0.01$ , time series smoothing weight $\lambda_{\text{smoot}h}=0.1$ , feedback utility weight $\lambda_{\mathrm{fb}}=0.5$ , task weight is set to $w_{p}=1.0,w_{e}=0.8,w_{v}=0.8$ , feedback gain γ=10.0 , output clipping interval is taken $u_{\min}=-1,u_{\max}=1$ . During the training process, batch normalization and layer normalization are used to maintain numerical stability of the hidden layer after each parameter update, and the training is rolled back at the lowest point of the validation set to retain the optimal weights.

The above training optimization and feedback generation process transforms cross-modal fusion time series predictions into executable personalized feedback signals through multi-task loss construction, constraints based on entropy and time series smoothing, a stable numerical update mechanism, and closed-loop feedback utility constraints. It also uses closed-loop evaluation to drive the adaptive fine-tuning of the model during training.

## 3 Experimental design and implementation

### 3.1 Experimental dataset construction

The study recruited 45 participants with varying levels of volleyball proficiency, including young professionals, university athletes, and amateur enthusiasts (see Table 3). The participants were aged between 15 and 28 years (Mean = 21.4, SD = 3.2), with heights ranging from 165 cm to 195 cm. Prior to the experiment, all participants (and legal guardians for those under 18) provided written informed consent. The experimental procedures regarding human subjects were strictly adhered to the Declaration of Helsinki and were approved by the institutional review board. During the experiment, the research team constructed a multi-layered dataset targeting individuals at different training levels to ensure sufficient motion diversity and sample coverage for the multimodal time series modeling process. The experimental subjects included young professionals, university teams, amateur clubs, middle school interest classes, and students from physical education colleges. Gesture capture, electromyography sensors, and accelerometers were used throughout the data collection process to continuously sample core volleyball training movements such as serving, spiking, blocking, passing, padding, and run-ups, and the cumulative training duration was recorded. To ensure the reliability of the subsequent model in motion recognition and load modeling, this section summarizes the experimental subjects, motion categories, and acquisition duration. The results are shown in Table 3.

**Table 3:** Distribution of experimental subjects and actions.

Group ID	Number of participants	Action categories	Total duration (minutes)
G1 (University Volleyball Team)	10	Spiking, Blocking, Approach Run	320
G2 (Amateur Volleyball Club)	8	Serving, Serve Reception, Setting	260
G3 (Physical Education Students)	12	Approach Run, Jumping, Spiking	340
G4 (Middle School Volleyball Class)	6	Setting, Passing, Blocking	210
G5 (Professional Youth Group)	9	Serving, Spiking, Blocking, Serve Reception	370
Total	45	6 Core Actions	1,500

All participants were within the age range of 15 to 28 years and their height ranged from 165 to 195 cm. Surface EMG sensors were placed on the skin overlying the bellies of the following major muscle groups involved in volleyball actions: biceps brachii, triceps brachii, anterior deltoid, pectoralis major, rectus femoris, and gastrocnemius. The specific combination of muscles monitored was adjusted per the focus of the training movement for each experimental group, ensuring coverage of primary movers for spiking, blocking, serving, and jumping actions. The distribution of experimental subjects showed that the professional youth group and physical education college students had the highest proportion of action collection time, reaching 370 and 340 min, respectively. This was primarily due to the stability and continuity of these groups’ movements during training, which reduced the number of invalid segments removed, effectively extending data retention. The university volleyball team’s training time was 320 min, indicating that their training intensity and frequency approached professional standards, but the movement continuity was slightly lower, resulting in incomplete data retention. The amateur club group’s collection time was 260 min, lower than that of the university and professional groups. This was due to insufficient standardization of movement execution, resulting in the rejection of some sequences due to substandard quality. The middle school interest class’s total time was 210 min, the lowest of all groups. This was primarily due to their lack of sports experience, resulting in frequent interruptions and unstable segments between movements, which reduced the proportion of valid data. Overall, differences in training proficiency and technical stability across different groups directly influenced the distribution of data collection time. For each subject and action sequence, the multimodal time-series data were sorted chronologically and split into training, validation, and test sets with a ratio of 70%/15%/15%. The earliest segment was used for training, the subsequent segment for validation, and the final segment for testing, ensuring that all test samples occurred strictly after the training data.

### 3.2 Experimental platform and implementation environment

During the experiment, the research team established a complete training environment based on a virtual simulation platform. They combined posture capture, electromyography, and accelerometers to collect multimodal data, ensuring the simultaneous acquisition of kinematic and physiological signals on the same platform. After data collection, a unified hardware and software environment was used for storage, modeling, and training, achieving a closed-loop support from virtual training scenarios to algorithm implementation. To clarify the experimental conditions and environmental configuration, the relevant parameters are shown in Table 4.

**Table 4:** Experimental environment parameters.

Platform/category	Configuration item	Parameter description	Version/specification
Virtual Simulation Platform	Engine Type	Unity 3D Engine	2021.3 LTS
Data Acquisition Device	Motion Capture System	Optical motion capture, 12 channels	100 Hz
Data Acquisition Device	EMG Sensor	Surface EMG acquisition, 8 channels	1,000 Hz
Data Acquisition Device	Accelerometer	Three-axis acceleration, range ±16 g	200 Hz
Hardware Environment	GPU	NVIDIA RTX 3080	10 GB VRAM
Hardware Environment	CPU	Intel Core i7-12700K	12 cores, 3.6 GHz
Hardware Environment	Memory	DDR4	32 GB
Software Environment	Deep Learning Framework	PyTorch	2.0.1
Software Environment	Programming Language and Toolchain	Python	3.9
Software Environment	Operating System	Ubuntu	20.04 LTS

This table summarizes the construction conditions of the virtual simulation platform, the sensor acquisition accuracy and number of channels, and the core hardware and software parameters, ensuring that experimental data is processed in an efficient and stable environment. The GPU and CPU configurations meet the training requirements of deep learning models, while the matching memory and operating system support the storage and access of large-scale time series data. The deep learning framework and programming environment lay the foundation for the implementation of DS-LSTM and temporal attention mechanisms.

### 3.3 Ablation study of model architecture components

To examine the roles of two-stream structures and temporal attention mechanisms in multimodal temporal modeling, this study conducted comparative modeling of different structural configurations while maintaining consistency in data partitioning, preprocessing procedures, network depth, hidden dimensions, and training strategies. The single-stream LSTM and the proposed DS-LSTM were implemented and trained under identical experimental conditions, including the same input features, data splits, network depth, hidden dimensions, optimizer, learning rate schedule, batch size, and stopping criteria, so that the comparison directly reflects architectural differences. Experimental setups included single-stream temporal modeling, modeling with only two-stream structures, modeling with only temporal attention, and a complete model employing both two-stream structures and temporal attention mechanisms. All models converged within the same number of training epochs, and action classification accuracy, electromyography load modeling error, and velocity curve fitting performance were evaluated on the same test set to observe the impact of structural changes on multimodal modeling behavior. The relevant results are summarized in Table 5.

**Table 5:** Ablation results of model components.

Model configuration	Motion accuracy (%)	EMG error (%)	Velocity R^2^
Single-stream LSTM	86.4	8.7	0.83
Dual-stream LSTM without attention	90.2	6.0	0.87
Attention-based single-stream LSTM	88.1	7.4	0.85
Dual-stream LSTM with temporal attention	93.1	3.8	0.91

The results in Table 5 show that the single-stream LSTM exhibits high error levels in action recognition and EMG load modeling, with an action classification accuracy of 86.4 and an EMG error of 8.7. This indicates that heterogeneous modalities generate significant information interference when modeled in a unified temporal space. When a two-stream structure is introduced without using an attention mechanism, the action accuracy improves to 90.2, the EMG error decreases to 6.0, and the velocity fitting coefficient increases to 0.87, indicating that temporal decoupling of kinematic and physiological signals makes the model’s representation of their respective dynamic modes more stable. The single-stream model using only temporal attention achieves an action accuracy of 88.1 and an EMG error of 7.4, showing that temporal weight allocation has a suppressive effect on redundant segments, but it is still limited by the modeling inconsistencies caused by feature mixing. When the dual-stream structure and time attention are introduced simultaneously, the motion accuracy is improved to 93.1, the electromyographic error is reduced to 3.8, and the velocity fitting coefficient reaches 0.91. This indicates that performing time weighting after completing intramodal temporal modeling effectively coordinates cross-modal dynamic information, thereby forming a stable and consistent modeling result.

## 4 Results analysis

### 4.1 Motion capture accuracy analysis

**Figure 3:**
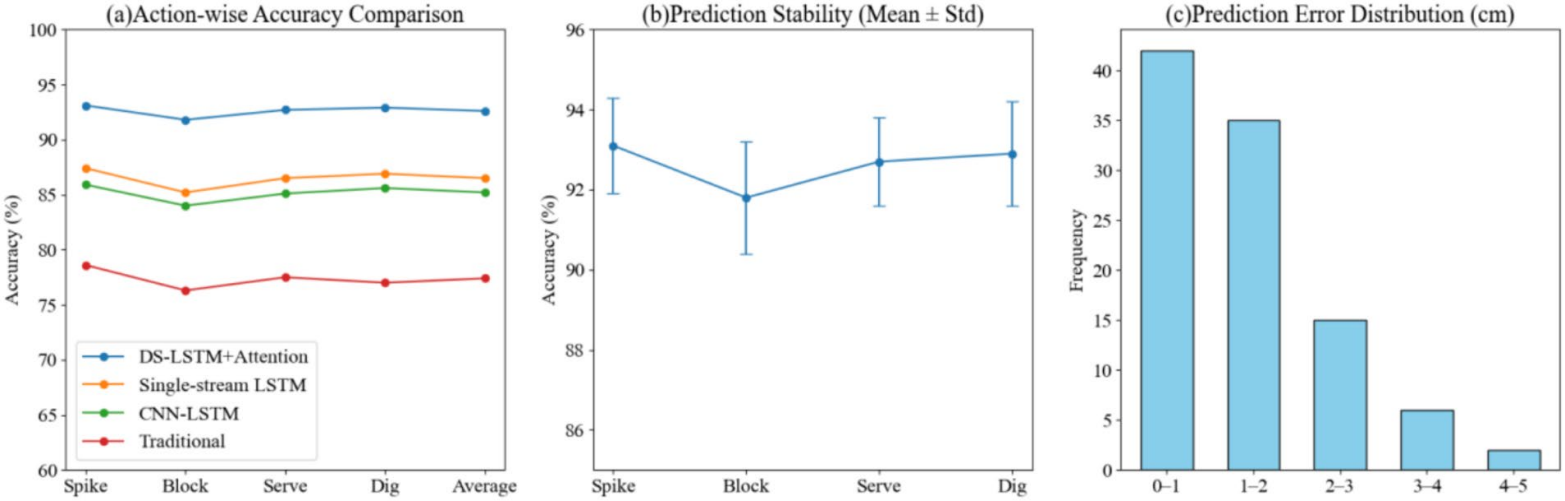
Motion capture accuracy and stability analysis comparing the proposed DS-LSTM against baseline methods. **(a)** Action-wise accuracy comparison showing the proposed method (blue) achieving higher accuracy in spike and block actions. **(b)** Prediction stability error bars indicating consistent performance. **(c)** Histogram of prediction error distribution, highlighting that 77% of errors fall within the minimal 0–2 cm range.

In the motion capture accuracy experiment, by constructing prediction results under different action categories, the performance of the method proposed in this paper is compared with other typical methods in the posture recognition task. The stability of the model in different actions is tested by combining multiple repeated experiments. At the same time, the statistical distribution analysis of the key point coordinate prediction error is carried out to fully reflect the dynamic performance of the model and the differences in data characteristics. The results are shown in Figure 3.

The data in the figure show that our method achieves 93.1% accuracy for the spike action, while traditional methods achieve only 78.6% for the same action. This difference stems from the dual-stream architecture’s superior ability to capture temporal dependencies in high-speed movements, thereby reducing transient loss. For the block action, the accuracy is 91.8%, compared to 85.2% for the single-stream LSTM. This higher accuracy is primarily due to the interaction of cross-modal features, which enables more complete temporal modeling of upper and lower limb coordination. In the stability analysis, the standard deviation of the serve action is only 1.1, less than the 1.4 for the block action. This is because the serve action pattern is relatively fixed and has high temporal repeatability, which makes the model converge more stably during learning. Error distribution statistics show that 77 cases, representing 77% of the total data, have prediction errors less than 2 cm. This concentration is related to the attention mechanism’s ability to filter critical time periods, effectively reducing the impact of non-critical movements on prediction accuracy. Overall, this method demonstrates consistent advantages in both accuracy and stability (Figure 3).

### 4.2 Muscle load modeling error analysis

**Figure 4:**
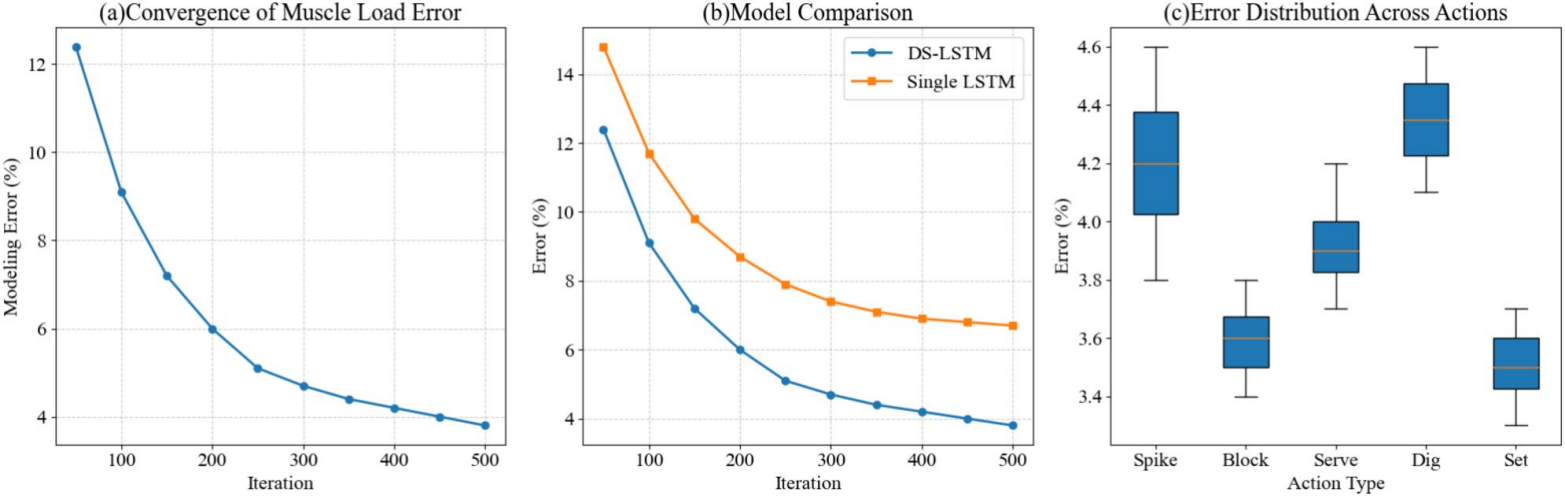
Analysis of EMG load modeling error convergence and motion-specific distribution. **(a)** The loss curve demonstrates rapid convergence to a 3.8% error rate after 500 iterations. **(b)** Model comparison showing the DS-LSTM (blue) reduces error more effectively than the single-stream LSTM (orange). **(c)** Boxplot of error distribution across varying volleyball actions, reflecting higher variability in dynamic movements like Digging.

In the EMG load modeling experiment, multimodal time-series data from volleyball-specific physical training was collected simultaneously using posture capture, EMG, and accelerometers. A dual-stream architecture was used to model kinematic and physiological characteristics separately, and a temporal attention mechanism was incorporated to achieve cross-modal fusion. During training, 500 iterations were set to monitor model error convergence. Comparative experiments were further conducted to verify the differences between the DS-LSTM and single-stream LSTM models. The error distributions of five typical volleyball movements under repeated experiments were statistically analyzed to assess movement-level stability and volatility. The results obtained based on this experimental process are shown in Figure 4.

Experimental results show that the overall convergence curve error dropped to 3.8 percent after 500 iterations, indicating that the model gradually overcomes high-frequency noise interference with continued training. This is because time alignment and filtering preprocessing suppress redundant signals, enabling the LSTM memory units to effectively capture the long-term dependencies of the EMG signals. In a model comparison, the DS-LSTM achieved an error of 6.0 percent after 200 iterations, while the single-stream LSTM achieved an error of 8.7 percent during the same period. This difference is primarily due to the dual-stream architecture’s separation of kinematic and physiological features, avoiding gradient convergence instability caused by feature aliasing. A boxplot of the action category errors shows that the median errors for Block and Set are 3.6 percent and 3.5 percent, respectively, while the median error for Dig reaches 4.3 percent, indicating that high-speed directional changes introduce EMG fluctuations, making accurate predictions in this category more difficult for the model. Overall, the multimodal fusion strategy offers advantages in reducing error levels and improving training data stability. The observed EMG load error patterns align with established EMG feature analysis practices, where lower errors correspond to consistent muscle activation timing and amplitude across trials, reflecting stable neuromuscular coordination during volleyball-specific movements.

### 4.3 Speed curve fitting analysis

**Figure 5:**
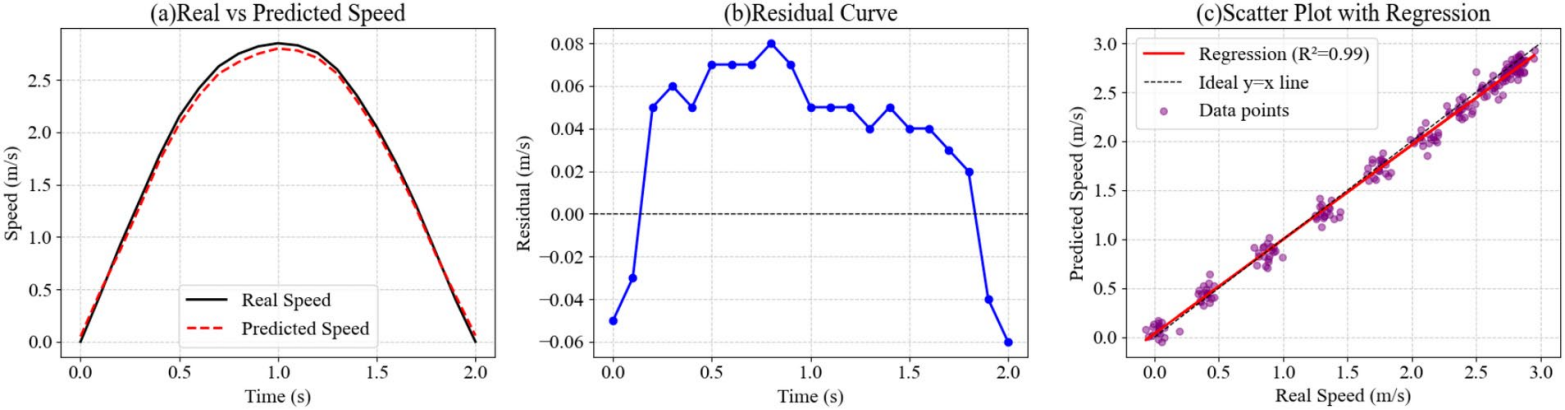
Velocity curve fitting analysis verifying model precision. **(a)** Comparison of Real (black) vs. Predicted (red) speed curves showing tight alignment with a peak deviation of only 0.05 m/s. **(b)** Residual curve highlighting minor deviations primarily during peak velocity phases. **(c)** Regression scatter plot (R^2^ = 0.91) confirming a strong linear correlation between predicted and actual velocities.

In this experiment, we used multimodal sensors to collect posture and velocity sequences during volleyball physical training. We then used the DS-LSTM model to fit and model the movement velocity to examine the discrepancies between the predicted results and the actual measurements. After normalization and time-series alignment, the raw data was input into the model to produce the predicted output. We then systematically evaluated the model’s fitting performance and error distribution characteristics by comparing the actual velocity curves with the predicted curves, analyzing the residual distribution, and performing regression scatter point fitting. The results are shown in Figure 5.

The results in the figure show that the true and predicted velocity curves closely match each other for most periods of time, reaching a peak between 1.0 and 1.5 s. The true maximum value is 2.85 meters per second, while the predicted value is 2.80 meters per second, a difference of 0.05 meters per second. This minor error is primarily due to transient fluctuations in the accelerometer during high-speed motion. The residual curve shows a positive deviation of 0.05 to 0.08 meters per second between 0.4 and 1.0 s, which is related to phase differences caused by synchronization errors in the electromyographic signals. In the scatter regression plot, the data points are distributed close to the diagonal, and the coefficient of determination calculated by the regression is 0.91, indicating that the model has strong fitting stability across different velocity ranges. In summary, this method can effectively characterize the dynamic characteristics of velocity during volleyball physical training and maintain high prediction accuracy.

### 4.4 Explanatory analysis of attention weight distribution

**Figure 6:**
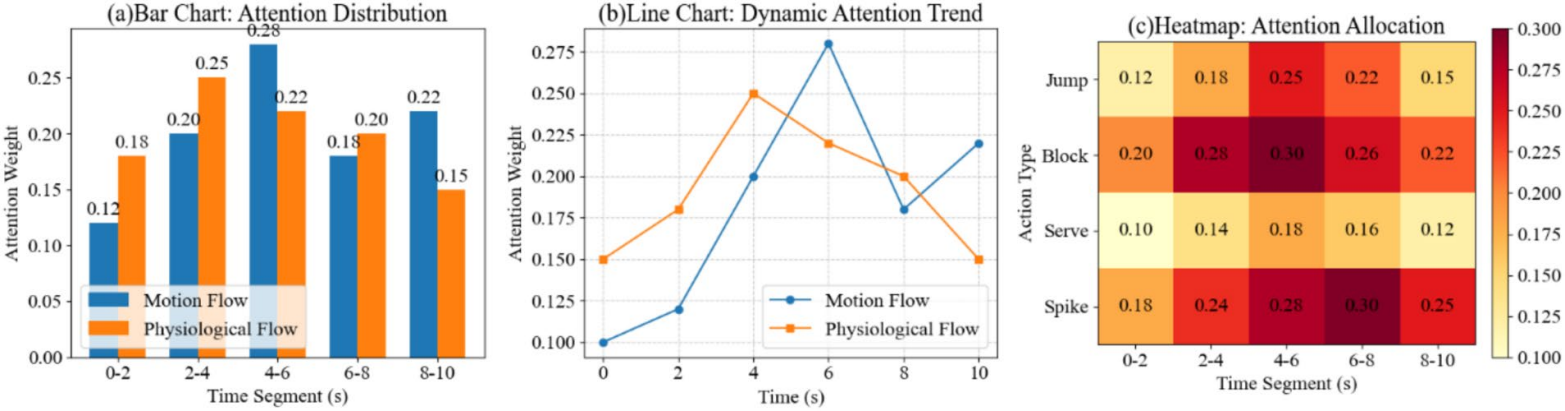
Spatiotemporal visualization of attention weight distribution. **(a)** Bar chart contrasting kinematic vs. physiological attention weights across time segments. **(b)** Line chart illustrating the dynamic shift in attention intensity during movement execution. **(c)** Heatmap showing how the model prioritizes specific time windows (e.g., 4–6 s) for different action types such as blocking and spiking.

In a virtual simulation experiment of volleyball physical training, to explore the model’s dynamic nature and cross-modal explanatory power in temporal feature selection, we conducted comparative modeling of the attention distribution of kinematic and physiological streams across different training time segments. This was further extended to a two-dimensional interactive analysis of movement categories and time segments. Based on multimodal time series data collected by posture, electromyography, and accelerometer sensors, the experiment extracted weight information for key time segments after two-stream LSTM encoding and attention weighting. The weight differences were then compared across kinematic and physiological features. Finally, multi-view diagrams of the model’s attention patterns across temporal segments and movement components were generated, including histograms, broken line trends, and heat matrix diagrams, as shown in Figure 6.

The results show that the kinematic stream’s attention weight for the 4–6 s period is 0.28, higher than the physiological stream’s 0.22. This phenomenon stems from the dominance of postural changes caused by the rapid jump during this period over the motion characteristics. The physiological stream’s weight for the 2–4 s period reaches 0.25, while the kinematic stream’s is only 0.20. This is primarily due to the increased muscle load early in training, which makes the EMG signal intensity more pronounced in driving the model weights. In the movement comparison, the Block’s weight for the 4–6 s period is 0.30, higher than the Spike’s 0.28 and the Jump’s 0.25. This result is due to the aggregation of physiological and kinematic signal characteristics generated by the combined upper limb force and core stability during the blocking process. Overall, the differences in weights over time and movement type indicate that the model’s attention to kinematic and physiological characteristics switches periodically during training.

### 4.5 Reliability analysis across trials and across subjects

To systematically evaluate the predictive consistency of the model across different trials and subjects, this experiment selected three key indicators: motion recognition accuracy, electromyographic load modeling error, and velocity fitting error. Based on data from all 45 subjects in multiple repeated trials, the intraclass correlation coefficient (ICC) and coefficient of variation (CV%) were calculated to quantify the model’s repeatability and stability. The relevant reliability statistics are shown in Table 6.

**Table 6:** Statistics of model prediction reliability indicators.

Evaluation metric	Action category	Subject group	ICC	CV (%)	*p*-value
Action Recognition Accuracy	Spike	All participants	0.94	3.2	>0.05
Action Recognition Accuracy	Block	All participants	0.92	3.8	>0.05
EMG Load Modeling Error	Serve	Professional group	0.91	4.1	>0.05
EMG Load Modeling Error	Dig	Amateur group	0.88	5.3	>0.05
Velocity Fitting Error	Approach Jump	University team	0.93	4.5	>0.05
Attention Weight Consistency	Spike	All participants	0.89	6.1	>0.05

The model exhibits high consistency in action recognition tasks, with ICC values of 0.94 and 0.92 for spiking and blocking actions, respectively, and CV% values below 4.0%, indicating that the dual-stream structure effectively extracts common temporal features of motion across different subjects. These results indicate that the proposed model maintains consistent prediction behavior at the individual subject level across repeated trials, demonstrating subject-specific stability rather than reliance on averaged population performance. In electromyography (EMG) load modeling, the professional group achieved an ICC of 0.91 and a CV% of 4.1% for serving, superior to the amateur group’s 0.88 and 5.3% for passing. This reflects the impact of technical standardization on EMG signal stability, with greater variability in action execution among amateur subjects leading to increased predictive volatility. Velocity fitting showed an ICC of 0.93 and a CV% of 4.5% for the approach jump, indicating strong generalization ability of the model to dynamic velocity curves. The consistency ICC of attention weights across trials was 0.89, demonstrating that the temporal attention mechanism stably captures key motion phases. Overall, the model demonstrates good cross-trial and cross-subject reliability across multiple tasks, making it suitable for training and analysis scenarios with diverse populations.

To facilitate a direct quantitative comparison between data-driven temporal modeling and handcrafted signal analysis, reliability metrics reported in this study were contrasted with representative sEMG and IMU feature-based results reported in prior musculoskeletal monitoring research. The comparison focuses on intraclass correlation coefficients and coefficients of variation, which were used in both studies to assess reproducibility and precision across repeated trials. The handcrafted feature results were drawn from a validated wearable-based rehabilitation study that evaluated time-domain and frequency-domain sEMG features together with IMU acceleration characteristics under controlled exercise protocols, while the DS-LSTM results were obtained from the multimodal volleyball training dataset analyzed in this work. The comparison is summarized in Table 7.

**Table 7:** Reliability comparison between DS-LSTM outputs and handcrafted sEMG–IMU features.

Method	Data type	ICC range	CV range (%)
Handcrafted sEMG–IMU features (Amin et al., 2025)	sEMG time and frequency features, IMU acceleration	0.81–0.98	5.7–14.4
DS-LSTM (this study)	Multimodal temporal outputs	0.88–0.94	3.2–6.1

The reliability metrics indicate that the DS-LSTM outputs exhibit a narrower variability range and higher consistency across trials compared with handcrafted sEMG and IMU features. The handcrafted feature-based analysis shows ICC values spanning from 0.81 to 0.98, accompanied by coefficients of variation extending up to 14.4 percent, reflecting sensitivity to signal amplitude fluctuations and feature extraction stability across repetitions. In contrast, the DS-LSTM results demonstrate ICC values concentrated between 0.88 and 0.94, with coefficients of variation remaining below 6.1 percent, indicating reduced dispersion in repeated measurements. This difference arises from the end-to-end temporal modeling of multimodal sequences, which suppresses trial-level signal variability through recurrent state propagation rather than relying on isolated signal descriptors. These results confirm that DS-LSTM-based modeling provides more stable reliability characteristics when evaluated using the same statistical validation criteria.

### 4.6 Analysis of training optimization feedback effects

**Figure 7:**
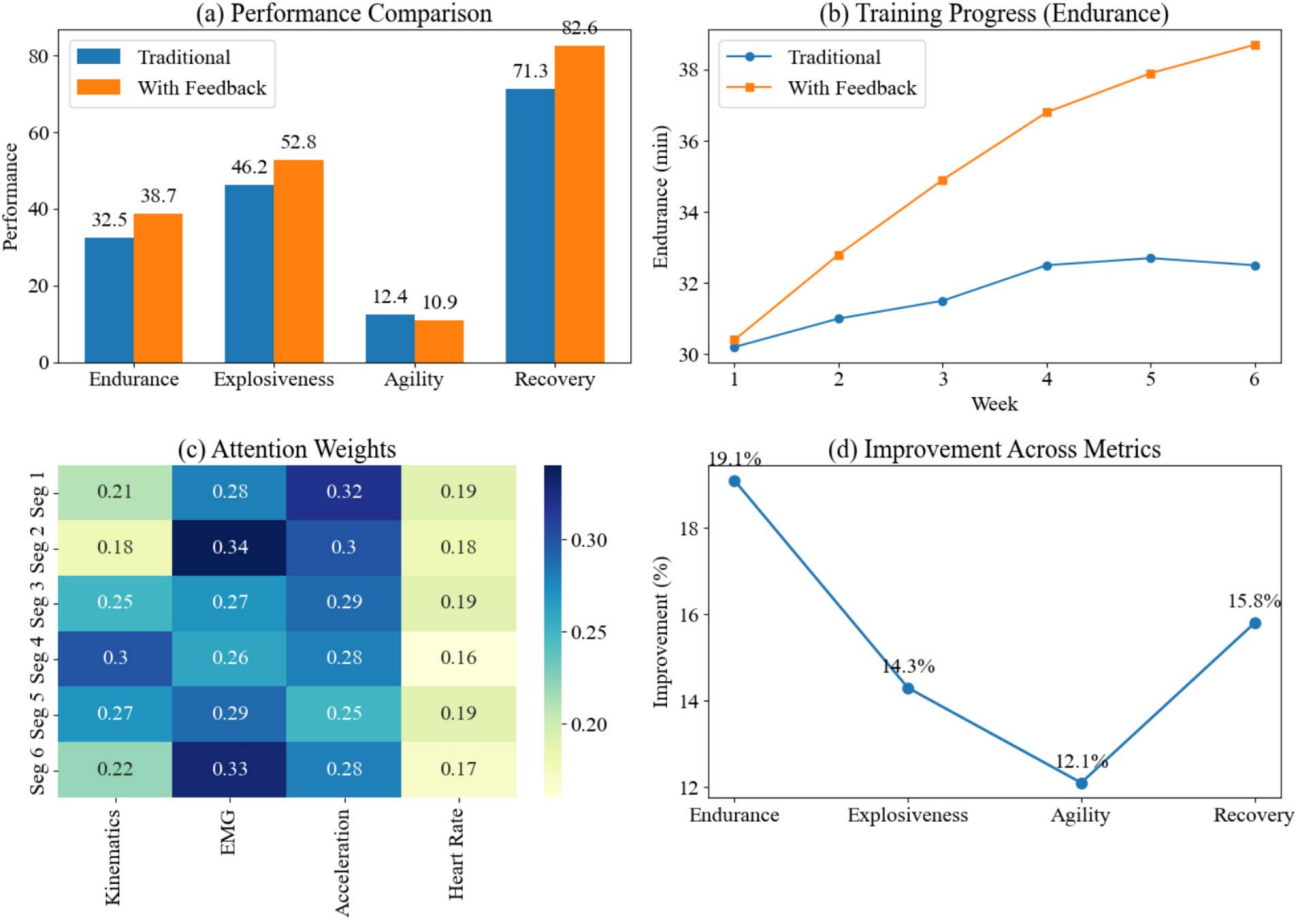
Evaluation of training optimization feedback effects. **(a)** Comparison of physical metrics (Endurance, Explosiveness, Agility, Recovery) between traditional and feedback-based training. **(b)** Endurance progress curve over 6 weeks showing steady improvement. **(c)** Heatmap of feature-specific attention weights. **(d)** Percentage improvement across all metrics, demonstrating the efficacy of the personalized feedback loop.

In this experiment, using a virtual simulation platform as support, we conducted a multi-dimensional dynamic comparison of traditional training and personalized feedback training. We focused on collecting four core metrics: endurance, explosive power, agility, and recovery efficiency. We tracked the temporal changes in athletic performance during training and, combined with the weight distribution of the attention mechanism output, revealed differences in the model’s focus on different features and training phases. This approach not only compared the final performance differences between the two training models, but also provided in-depth analysis of the data across both the temporal and feature dimensions, ultimately yielding the results shown in Figure 7.

As shown in the results, endurance reached 38.7 min with personalized feedback training, compared to 32.5 min with traditional training. This improvement is primarily attributed to the dynamic adjustment of load distribution during training, which reduces the rate of lactate accumulation and delays the onset of fatigue. Explosive power reached 52.8 cm with feedback training, a significant improvement compared to 46.2 cm with traditional training. This result is related to modifications in joint angle control and muscle activation patterns. Sensitivity decreased to 10.9 s with feedback training, compared to 12.4 s with traditional training. This is due to the model’s increased focus on acceleration features during high-intensity transitions, which improves the latency of movement responses. Recovery efficiency increased to 82.6% with feedback training, compared to 71.3% with traditional training. This is due to more effective control of heart rate fluctuations, which enhances the recovery capacity of the autonomic nervous system. Overall, the personalized feedback mechanism, through optimized load distribution and feature focus strategies, leads to comprehensive improvements in athletic performance.

## 5 Conclusion

This paper proposes a dynamic modeling method for volleyball physical training based on the DS-LSTM and temporal attention mechanism. By collecting multimodal time series data using posture capture, electromyography, and accelerometers, and performing time alignment and feature fusion, this method achieves intelligent prediction of training status and personalized feedback. Experimental results show that this method achieves 93.1% accuracy in spike motion recognition, significantly exceeding the 78.6% of traditional methods, demonstrating the advantages of cross-modal interaction in high-speed motion modeling. In muscle load prediction, the error drops to 3.8% after 500 iterations, surpassing the 8.7% of a single-stream LSTM. This demonstrates the significant effectiveness of the dual-stream architecture in stabilizing convergence and reducing noise. In velocity curve fitting experiments, the predicted peak value of 2.80 meters per second differs only 0.05 meters per second from the true value of 2.85 meters per second, with a coefficient of determination of 0.91, demonstrating the model’s high fitting accuracy during dynamic motion. Research shows that combined with the feedback mechanism of the virtual simulation platform, the trainees have achieved significant improvements in endurance, explosive power and sensitivity. This shows that this method not only breaks through the limitations of traditional training that relies on experience and judgment, but also shows strong interpretability and scientificity in multimodal feature interaction and key period screening, providing reliable support for the personalized optimization of volleyball physical training and the promotion and application of virtual simulation teaching.

Despite the promising results, this study has certain limitations that outline directions for future research. First, the current dataset focuses primarily on specific age groups and skill levels; future work will expand the sample diversity to include a wider range of demographics to enhance model generalization. Second, while the current system operates effectively in a simulation environment, future iterations will focus on optimizing the algorithm for edge computing devices to minimize latency in real-world, on-court training scenarios. Finally, we plan to extend this dual-stream attention framework to other complex team sports, such as basketball and football, to validate its cross-disciplinary applicability.

## Data Availability

The original contributions presented in the study are included in the article/supplementary material, further inquiries can be directed to the corresponding author.
